# Effect of Nitrogen Doping on the Optical Bandgap and Electrical Conductivity of Nitrogen-Doped Reduced Graphene Oxide

**DOI:** 10.3390/molecules26216424

**Published:** 2021-10-25

**Authors:** Gunawan Witjaksono, Muhammad Junaid, Mohd Haris Khir, Zaka Ullah, Nelson Tansu, Mohamed Shuaib Bin Mohamed Saheed, Muhammad Aadil Siddiqui, Saeed S. Ba-Hashwan, Abdullah Saleh Algamili, Saeed Ahmed Magsi, Muhammad Zubair Aslam, Rab Nawaz

**Affiliations:** 1 BRI Institute, Jl. Harsono RM No. 2, Ragunan, Jakarta 12550, Passsar Minggu, Indonesia; 2 Department of Electrical and Electronic Engineering, Universiti Teknologi PETRONAS, Seri Iskandar 32610, Perak, Malaysia; harisk@utp.edu.my (M.H.K.); zaka_18000817@utp.edu.my (Z.U.); adil_siddiqui00@yahoo.com (M.A.S.); saeedsb2013@gmail.com (S.S.B.-H.); abdullah_17008405@utp.edu.my (A.S.A.); saeed_19001716@utp.edu.my (S.A.M.); m.zubair_g02974@utp.edu.my (M.Z.A.); 3 Department of Electronic Engineering, Balochistan University of Information Technology, Engineering and Management Sciences, Quetta 87300, Balochistan, Pakistan; 4 School of Electrical and Electronic Engineering, The University of Adelaide, Adelaide, SA 5005, Australia; nelson.tansu@adelaide.edu.au; 5 Institute for Photonics and Advanced Sensing, The University of Adelaide, Adelaide, SA 5005, Australia; 6 Department of Mechanical Engineering, Universiti Teknologi PETRONAS, Seri Iskandar 32610, Perak, Malaysia; Shuaib.saheed@utp.edu.my; 7 Department of Fundamental Science, Universiti Teknologi PETRONAS, Seri Iskandar 32610, Perak, Malaysia; rabnawaz.utp@gmail.com or

**Keywords:** optical bandgap tunning, optoelectronic, nitrogen-doped reduced graphene oxide, conductivity

## Abstract

Graphene as a material for optoelectronic design applications has been significantly restricted owing to zero bandgap and non-compatible handling procedures compared with regular microelectronic ones. In this work, nitrogen-doped reduced graphene oxide (N-rGO) with tunable optical bandgap and enhanced electrical conductivity was synthesized via a microwave-assisted hydrothermal method. The properties of the synthesized N-rGO were determined using XPS, FTIR and Raman spectroscopy, UV/vis, as well as FESEM techniques. The UV/vis spectroscopic analysis confirmed the narrowness of the optical bandgap from 3.4 to 3.1, 2.5, and 2.2 eV in N-rGO samples, where N-rGO samples were synthesized with a nitrogen doping concentration of 2.80, 4.53, and 5.51 at.%. Besides, an enhanced n-type electrical conductivity in N-rGO was observed in Hall effect measurement. The observed tunable optoelectrical characteristics of N-rGO make it a suitable material for developing future optoelectronic devices at the nanoscale.

## 1. Introduction

The heteroatom atom doping of carbon-based nanomaterial, i.e., graphene, graphene oxide, and carbon nanotube, among others, has gained great attention in material science and research [1]. The unique and stable physicochemical properties of graphene with a low-cost synthesis at a large scale make it the most promising material for future optical and electronic applications [2]. The bandgap tunability of GO in the visible range could allow it to be used in mid-IR photodetectors and ultrafast lasers as a saturable absorber, potentially outperforming graphene [3]. In recent studies, certain extrinsic characteristics of chemically modified graphene have been investigated in several applications such as sensors [4], energy harvesting [5], supercapacitors [6], field-effect transistors [7], and solar cells [8]. Certain optical and electronic properties of graphene, and modified graphene-based material, such as chemical stability, optical saturation, high charge carrier mobility, transparency, and intrinsic zero bandgap nature with tunable bandgap ability, enable graphene-based materials to perform efficiently for the development of forthcoming optoelectronic devices [9]. However, pristine graphene is a zero-bandgap material, where the energy bandgap can be manipulated for several applications, including photolytic activities and solar cells [10].

In its intrinsic form, graphene is nearly transparent, with a lower light absorption coefficient and extreme thickness. When graphene is utilized as light active material in solar cell applications, it appears to have low-efficiency energy conversion compared with conventional solar cells [11]. Therefore, a modified graphene layer with a well-tuned and wide energy bandgap is required to enhance the light absorption coefficient, which is a huge task, but with tremendous interest [12]. More interestingly, an optical bandgap can be induced in graphene through chemical substitutional doping, where certain heteroatoms can be introduced into the graphene layer to tune and manipulate the related surface chemistry. In recent studies, different heteroatoms have been utilized for doping of graphene to tune the energy bandgap and enhance electrical conductivity, where oxygen [13], boron [14], and nitrogen [15] were commonly reported. Nitrogen and boron atoms are more favorable because they significantly modify graphene’s optical and electronic properties owing to similar atomic radii to carbon. Moreover, nitrogen and boron can induce interesting electrochemical properties while keeping a stable graphene structure [16].

Nitrogen as a dopant is the most widely investigated element for modifying the optical and electronic properties of graphene or reduced graphene. Nitrogen-related defect sites induced in graphene enhance electrochemical activity in graphene and reduce graphene oxide [17]. Besides, nitrogen as a dopant element in graphene can induce DOS (density of states) near the Fermi energy level and an energy bandgap opening. Nitrogen as a dopant in graphene also tends to produce electron-donor or acceptor states that can induce *p*-type or n-type semiconducting behavior, depending on the bonding configuration. Particularly, lower charge carrier mobility and electrical conductivity were reported in nitrogen-doped graphene compared with intrinsic graphene, which is attributed to the existence of nitrogen contents and the induced defect in pristine graphene. However, nitrogen doping shows the capability to function at the scattering center, which hinders the hole and electron transportation [18].

Nitrogen-doped reduced graphene oxide (N-rGO) is produced by chemical substitutional doping of graphene oxide with nitrogen as a dopant element. N-rGO shows ideal physicochemical properties for optoelectronic design applications such as higher chemical stability and large surface area. Optical and electronic properties can also be potentially optimized by bonding configuration [19]. Several nitrogen bonding configurations in N-rGO have also been reported, such as pyrrolic-N [19], pyridinic-N [20], oxide-N [21], and quaternary-N [22]. Different atomic percentage (at.%) of nitrogen [23] and each configuration produced a different effect in terms of charge carrier concentration and energy band structure [24]. 

The N-rGO synthesis is still fascinating and challenging, where the whole process has not been well understood and needs further exploration. The optimized synthesis of N-rGO with a certain level of control over doping concertation and the formation of bonding configuration is still lacking. Previously, different synthesis techniques have been employed for N-rGO syntheses, such as thermal annealing [24], plasma treatment [25], arc discharge [26], and chemical vapor deposition (CVD). The CVD synthesis approach has often been recommended as it is easy to scale up and yields comparatively high-quality N-rGO. The CVD synthesis approach involves several influential factors such as temperature, type of carrier gas, and nitrogen precursor (liquid, solid, or gas), which significantly influence the nitrogen concertation and physicochemical properties [27,28]. For instance, Lam et al. [29] used pyridine and dimethylformamide as liquid nitrogen precursors, with the former obtaining a low nitrogen concentration of about 0.64%. When liquid nitrogen is utilized as a precursor in CVD synthesis, it has the disadvantage of being expensive, hazardous, and highly flammable. Solid nitrogen percussors are an alternative to liquid phase nitrogen percussors, where solid precursors such as urea [24,30], pentachloro pyridine [31], monoethanolamides [32], and 1,3,5-triazine [20] have also been reported with higher nitrogen doping concertation from 4.4 to 7.5% [31].

**Figure 1:**
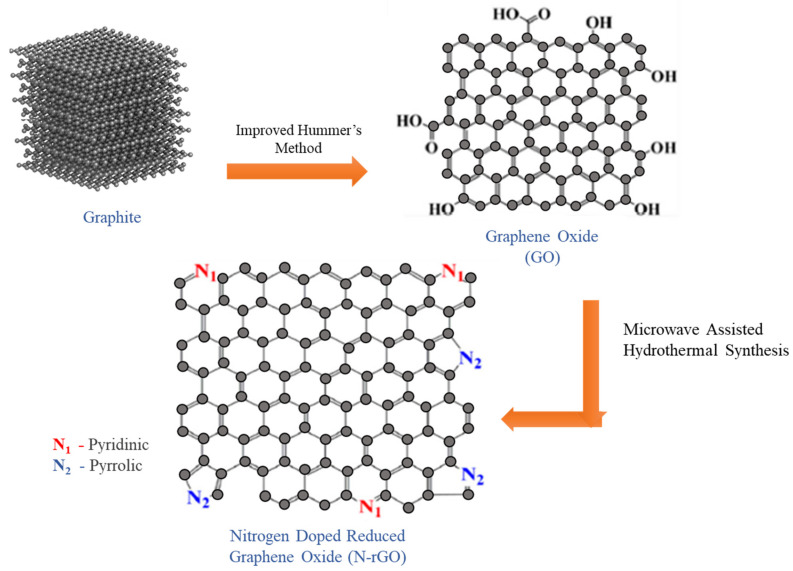
Microwave-assisted hydrothermal process for the synthesis of nitrogen-doped reduced graphene oxide.

Microwave-assisted and hydrothermal reduction synthesis of nitrogen-doped graphene has been demonstrated in several applications, such as electrode design, supercapacitor, lithium-ion batteries, and field-effect transistors [33]. In this study, microwave-assisted hydrothermal synthesis of N-rGO as a novel approach has been demonstrated for the tunable optical bandgap and enhanced electrical conductivity. The current study aimed to tune up the optical bandgap and electrical conductivity of N-rGO via manipulating the atomic percentage of nitrogen doping. Firstly, GO was synthesized as a starting material for the synthesis of N-rGO. The synthesis process of N-rGO is schematically illustrated in Figure 1. The morphological and structural properties of GO and N-rGO were determined using various analytical techniques including FESEM, Raman XRD, FTIR, and XPS. Hall effect measurement was performed to determine the electrical conductivity, where the optical bandgap study was performed via UV/vis absorption spectra and Tauc plot calculations.

## 2. Results and Discussions 

### 2.1. FESEM Analysis 

**Figure 2:**
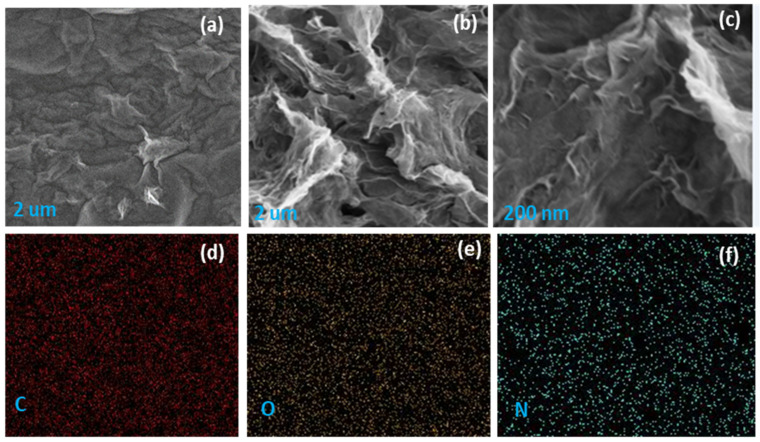
FESEM images of (**a**) GO and (**b**) N-rGO; (**c**) higher magnifications image of N-rGO; and element mapping of N-rGO (**d**) carbon (**e**) oxygen, and (**f**) nitrogen.

The field emission scanning electron microscopic (FESEM) images of GO and N-rGO and elemental mapping of N-rGO are shown in Figure 2. Several wrinkles and corrugation can be observed, possibly ascribed to oxygen-related functional groups and nitrogen dopant elements. Figure 2b shows that the wrinkles were more pronounced in N-rGO, indicating the effect of nitrogen doping on its morphology. Besides, Figure 2d–f present the element mapping of carbon, oxygen, and nitrogen of N-rGO, where homogeneous and regular dispersion of each element in the N-rGO sample was observed in a 2 µm^2^ selected area. FESEM analysis and EDX mapping revealed that nitrogen doping significantly affects the morphology and the homogeneous distribution of the elements, as shown in Figure 2d–f.

### 2.2. Raman Spectroscopy Analysis 

**Figure 3:**
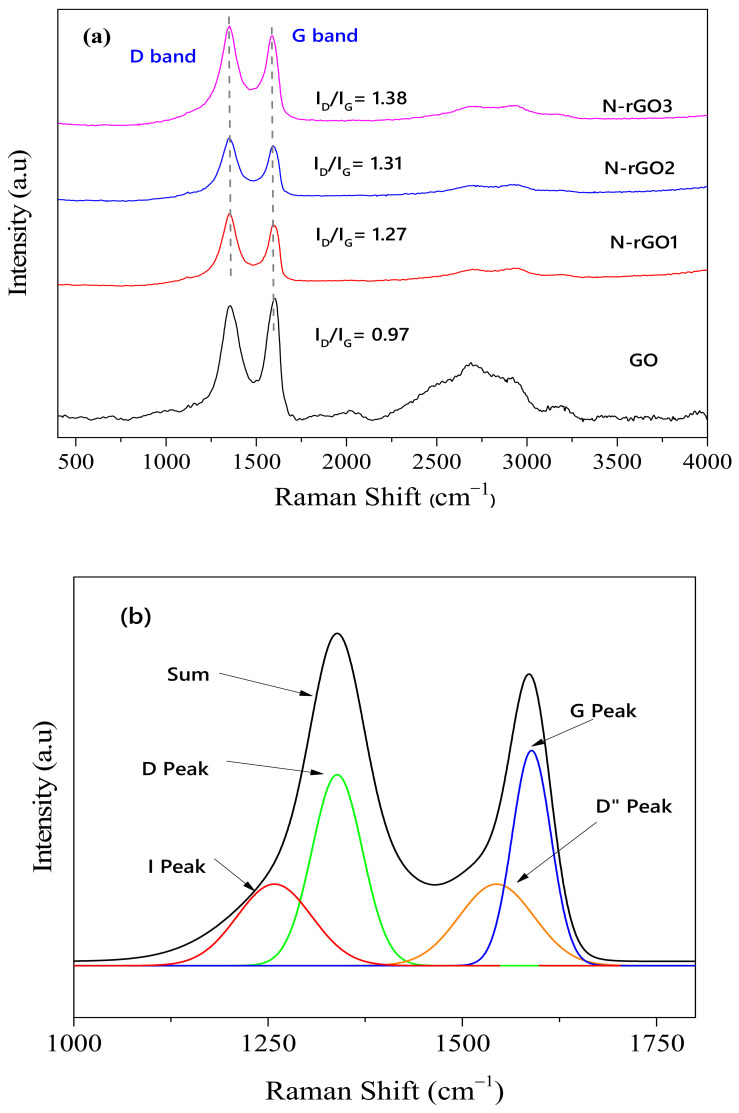
(**a**) Raman spectra of GO, N-rGO1, N-rGO2, and N-rGO3 and (**b**) deconvoluted Raman spectra of N-rGO3.

Raman spectroscopic analysis is an effective and valuable technique for evaluating the degree of disorder in nanomaterials [15]. Figure 3 shows a comparison of the Raman spectra of GO and N-rGO samples. The Raman spectrum of GO shows two major Raman peaks at 1348 cm^−1^ and 1600 cm^−1^, which can be assigned to the D and G band, respectively. The D band intensity peak at ~1348 cm^−1^ found in the GO spectra was ascribed to the disorder and defects in the graphite lattice owing to the presence of oxygen-related functional groups [14]. Most importantly, the Raman bands corresponding to second-order D, also known as 2D or G′, emerged at 2685 cm^−1^, ascribed to the phase of the two second-order phonon vibrations [34]. After N doping of GO, the two successive peaks related to the D (structural defects) and G (graphitic lattice) bands were observed at around 1200 and 1600 cm^−1^, respectively, corresponding to the vibrational scattering of E_2g_ symmetry in the graphitic lattice.

Furthermore, an increase in the D band intensity peak compared with the G band was examined in the Raman spectra of N-rGO. The intensity peak ratio (I_D_/I_G_) describes the nanomaterial quality and a corresponding concentration of SP^3^ defects in the SP^2^ hybridized graphene layer [35]. The increase in the I_D_/I_G_ ratio of N-rGO samples was examined, i.e., GO (0.97), N-rGO1 (1.27), N-rGO2 (1.31), and N-rGO3 (1.38). Moreover, an increment in the (I_D_/I_G_) ratio indicates the reduction of GO and the existence of nitrogen content owing to the extra scattering effect induced by electron doping [15]. The redshift of graphitic disorder (G + D) intensity peaks at 2950, 2965, and 2967 cm^−1^ in N-rGO1, N-rGO2, and N-RGO3 samples indicates the formation of few-layered graphene [15]. 

The Raman spectra of N-rGO was deconvoluted into four peaks, i.e., I peak (~1200 cm^−1^), D peak (~1358 cm^−1^), D″ peak (~1555 cm^−1^), and G peak (~1598 cm^−1^). Figure 3b shows a typical deconvoluted Raman spectrum for N-rGO3. The deconvoluted Raman spectra for N-rGO1 and N-rGO2 is included in the Supplementary Materials (Figure S1). The significance of D and G peaks has already been well addressed in previous reports [36,37], whereas the D″ peak and I peak have not been well addressed. The I peak was observed as a shoulder to the D peak, which is ascribed to the nitrogen-doped carbon structure [38] or a highly disordered carbon [39]. Particularly, an increase in nitrogen doping concentration resulted in a decrease in the calculated area of the I peak, as shown in the Supplementary Materials (Table S1). Sharifi et al. mentioned that the D″ peak increases with an increment in interlayer distance, in contrast with Vollebregt et al., who reported that the D″ peak decreases with an increment in crystallinity. The additional defect peak D′ appeared as a shoulder to the G peak [40,41]. Raman spectroscopic analysis of N-GO samples shows the dependence of the D″ and I peak on the nitrogen doping concentration. 

### 2.3. FTIR Analysis 

**Figure 4:**
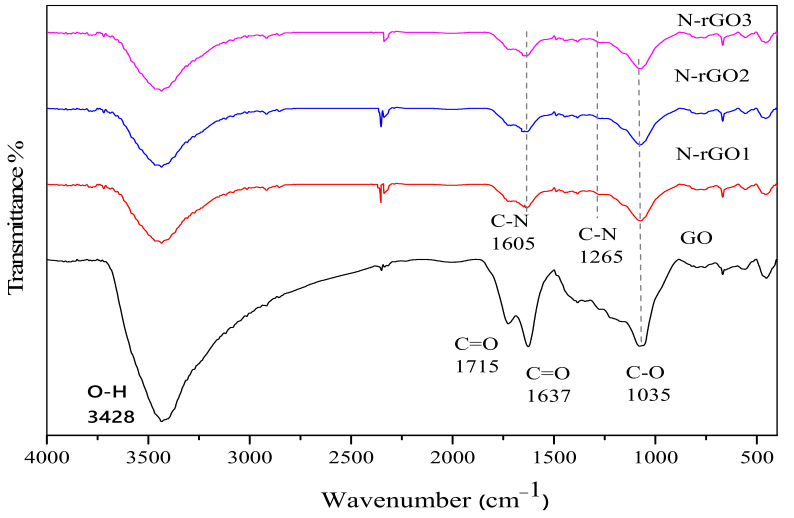
FTIR spectra of GO, N-rGO1, N-rGO2, and N-rGO3.

GO and N-rGO were also analyzed using FTIR spectroscopy to determine the functional groups present in the sample after oxidation and microwave-assisted hydrothermal reactions. The FTIR spectra of GO and N-rGO samples are presented in Figure 4. In the FTIR spectra of GO, a broad-spectrum peak in the range from 3000 to 3700 cm^−1^ was ascribed to the existence of a hydroxyl group and surface adsorbed water [42,43].

The other peaks at 1715, 1415, and 1227 cm^−1^ were ascribed to a ketone (C=O), carboxyl (COOH), and the epoxide (C–O–C) groups, respectively [44,45]. Moreover, the peak at around 1035 cm^−1^ can be assigned to the C–O groups stretching vibrations [46]. In the microwave-assisted hydrothermal reaction of GO and nitrogen source, the peak intensities of the oxygen-related group were significantly reduced, and some even disappeared. In FTIR spectra of N-rGO, a couple of new peaks appeared in the range of 1000 to 1700 cm^−1^. Particularly, in N-rGO synthesis process, the broad spectrum (^−^OH) stretch was reduced to a great extent, and N-C-related peaks were discovered at 1605 and 1265 cm^−1^, which are in good agreement with the reported values [47].

### 2.4. XPS Analysis 

**Figure 5:**
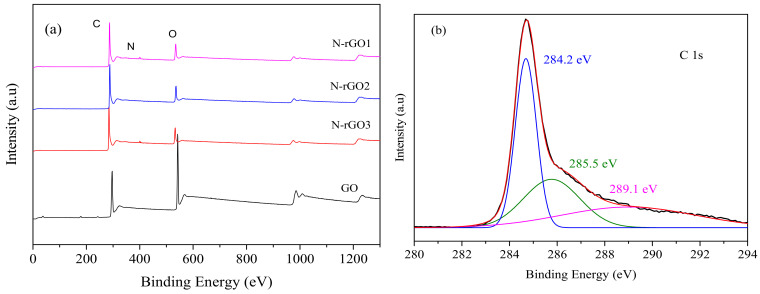

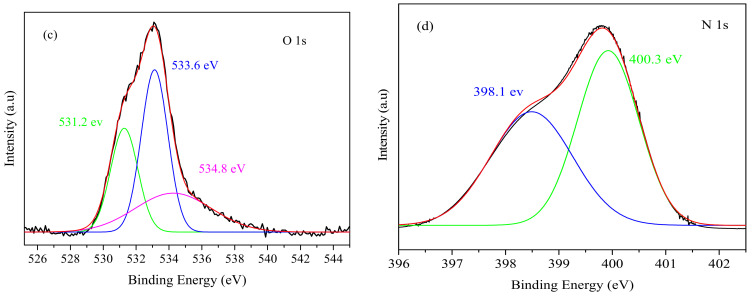
(**a**) XPS survey spectra of GO and N-rGO, and high resolution deconvoluted XPS spectra of N-rGO3 (**b**) C1s, (**c**) O1s, and (**d**) N1s. In addition, the atomic percentage of each element was determined from the XPS analysis of GO and N-rGO samples, listed in Table 1. The atomic percentage of nitrogen in N-rGO1, N-rGO2, and N-rGO3 samples was determined to be about 5.51, 4.53, and 2.80 at.%, respectively, which were consistent with theoretical values [49]. Each functional group identified in the XPS analysis of GO and N-rGO is also summarized in Table 2.

Figure 5a illustrates the X-ray photoelectron spectroscopy (XPS) survey scan of GO and N-rGO. In the XPS spectra of N-rGO samples, O 1s, C 1s, and N 1s appeared at 285.3, 533.5, and 400.1 eV, respectively. When the XPS spectra of GO and N-rGO were compared, a substantial decrease in O 1s was observed in the N-rGO spectrum, which was ascribed to the reduction in graphene oxide. The Gaussian deconvolution of O 1s, C 1s, and N 1s of corresponding peaks was also performed to further analyze the existence of functionalized states in N-rGO samples, as shown in Figure 5b–d. Figure 5b illustrates the deconvoluted C 1s XPS spectra of N-rGO, where several peaks were observed, including C=C (284.2 eV), C–OH (285.5 eV), and C=O (289.1 eV), associated with chemical bonding of carbon–carbon and C-oxygen-related functional groups. A strong peak in C 1s spectra at 284.2 eV was observed and ascribed to the SP^2^ bonding of carbon atoms, which indicates the existence of the majority of carbon atoms in the honeycomb structure [34]. In O 1s deconvolution spectra (Figure 5c), the corresponding peaks at 531.2, 533.6, and 534.8 eV were ascribed to the O=C–OH, C–OH, and C–O functional groups, respectively [48]. Moreover, the N 1s spectrum was also convoluted into two peaks, 398.2 and 400.3 eV, as shown in Figure 5d, which corresponds to the existence of pyridinic and pyrrolic-N graphitic structures [15].

### 2.5. XRD Analysis 

**Figure 6:**
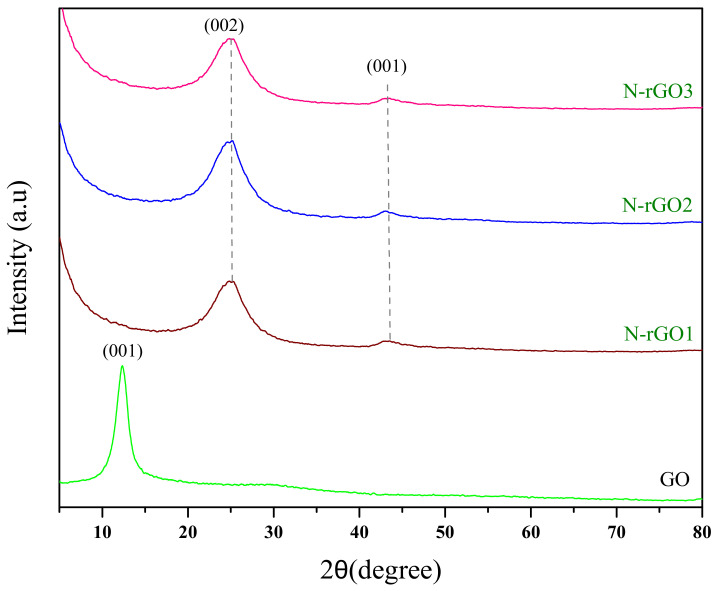
XRD patterns of GO, N-rGO1, N-rGO2, and N-rGO3 samples.

Figure 6 illustrates the X-ray diffraction patterns of GO and N-rGO samples. In the case of GO, a strong diffraction peak at 2θ = 11° was observed, which was assigned to the (001) plane. In the XRD patterns of N-rGO, the corresponding peak (001) almost disappeared, and new diffraction peaks (002) appeared at 2θ = 25.91°, 25.93°, and 26.1° in the XRD patterns of N-rGO1, N-rGO2, and N-rGO3, respectively [49]. Besides, I reduced XRD peaks (001) were also observed at 43° for all N-rGO samples.

The interlayer distance of the GO and N-rGO crystalline plane was obtained using Bragg′s law, as given in Equation (1) [50].

(1) $$\lambda =\frac{2dsin\left(\theta \right)}{n}$$

where *d*, *θ*, *n*, and *λ* are the interlayer distance, scattering angle, order of reflection, and wavelength of the incident X-ray source (1.54 Å), respectively. The interlayer distance for GO was determined to be about ~8.05 Å, which shows an agreement with [51,52]. Furthermore, a decrease in the interlayer distance was observed at about 4.26 Å, 4.10 Å, and 4.05 Å for N-rGO1, N-rGO2, and N-rGO3 samples, respectively. The higher interlayer distance of GO was attributed to the existence of oxygen-related functional groups, where the decrease in the interlayer distance N-rGO samples was ascribed to the reduction of functional groups and the existence of nitrogen contents [53]. 

The XRD peak intensities (002 planes) were increased with an increase in the doping concentration of nitrogen. The difference in peak shape reflects overlapped N-rGO sheets along the c-axis [54] and higher sample crystallinity [55]. The increase in the peak shape points to overlapped N-rGO sheets along the c-axis [54] and higher sample crystallinity [55]. This agrees with the calculated crystallite size along the c-axis (L_c_) (Table 3), which was derived using Scherer’s Equation and a dimensionless form factor of K = 0.90 [56]. The XRD analysis shows that the N-rGO3 is more crystalline and has a large Lc as compared with the N-rGO1 and N-rGO2 samples.

### 2.6. Hall Effect Measurement

The Hall effect measurements of GO and N-rGO samples were carried out at 300 K, in order to examine electrical conductivity, charge carrier mobility, and carrier type, as summarized in Table 4. An enhanced n-type conductivity was observed in N-rGO samples. In contrast, GO depicts relatively lower electrical conductivity as compared with the N-rGO material samples. This is because of the existence of higher oxygen-related groups, which makes the GO an insulating material. The amorphous structure of GO leads to distortions in SP^2^ bonding configuration owing to the SP^3^ bonding formations, i.e., COOH, C–O–C, and C–OH, which cause the random dispersions [15]. The isolation of the SP^2^ hybridized ring from the SP^3^ hybridized ring in the GO structure also prompts the insulation behavior. The enhanced electrical conductivity in N-rGO1, N-rGO2, and N-rGO3 samples was recorded to be ~0.625, 0.757, and 0.781 S/cm, respectively, where *p*-type electrical conductivity of GO was measured to be about 1.567 × 10^−6^ S/cm. Therefore, enhanced electrical conductivity in N-rGO samples was ascribed to the further reduction of graphene oxide, increment in SP^2^ related domains, and existence of nitrogen doping concentration. In addition, the pyrrolic-N bonding configuration in N-rGO material samples was more dominant in XPS analysis, which enhances the π-bond in carbon atoms by reducing the Stone–Wales effect and forming percolation ways for electrons, which ultimately results in quitting a conduction gap and enhancing n-type electrical conductivity.

### 2.7. UV/vis Analysis

**Figure 7:**
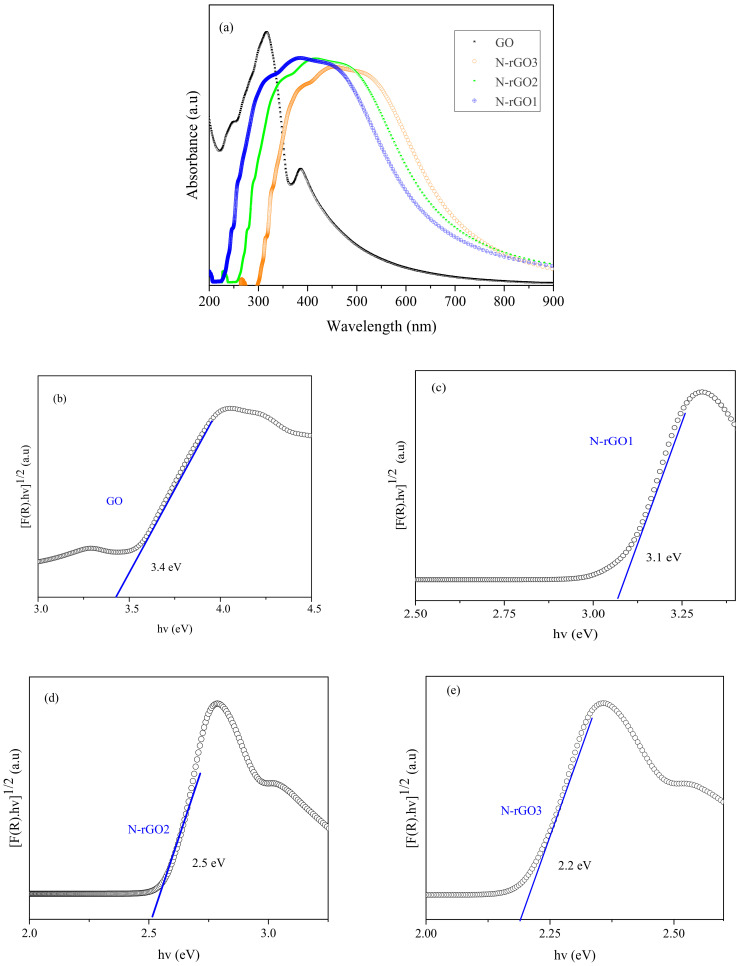
(**a**) UV/vis absorption spectra of GO, N-rGO1, N-rGO2, and N-rGO3; Tauc′s plots of samples (**b**) GO, (**c**) N-rGO1, (**d**) N-rGO2, and (**e**) N-rGO3.

The optical bandgap of GO and N-rGO samples was determined through ultraviolet−visible (UV/vis) spectroscopy analysis. The UV/vis absorption spectra of GO and N-rGO-based thin film (deposited on a glass substrate) are illustrated in Figure 7a. The UV/vis absorption spectra of GO, N-rGO1, N-rGO2, and N-rGO3 showed the absorption peaks at 364, 405, 495, and 564 nm, respectively, which correspond to a direct tunable optical bandgap from 3.4 to 2.2 eV. Notably, all nitrogen-doped samples depict light absorption spectra in the visible region. The lower light absorption of N-rGO samples at lower wavelength (UV) regions compared with GO indicates the restoration of the SP^2^ domains in the graphene sheet. In contrast, an additional absorption shoulder in the absorption spectrum of N-rGO samples (from 500 nm) can be ascribed to the n to π* related transitions. The n–π* related transitions also reveal the existence of pi* states and the homogeneous distribution of nitrogen atoms in N-rGO material samples.

The Tauc plot calculation for GO and N-rGO samples is presented in Figure 7b–d, where the direct optical bandgap for GO, N-rGO1, N-rGO2, and N-rGO3 was determined to be about 3.4, 3.1, 2.5, and 2.2 eV, respectively. The N-rGO3 sample with maximum nitrogen (~5.51 at.%) shows the lowest optical bandgap about 2.2 eV. It was also observed that, as the nitrogen concentration increases, the optical bandgap of N-rGO decreases. The decrease in optical bandgap can be attributed to the formation of compensating energy states that arise owing to the nitrogen dopant elements, consequently shifting the conduction band edges in N-rGO [57].

## 3. Materials and Methods

### 3.1. Materials

Graphite flakes (about 50 μm), ethanol (95.0%), sulfuric acid (H_2_SO_4_, 97%), potassium permanganate (KMnO_4_, 99.5%), phosphoric acid (H_3_PO_4_, 85%), hydrochloric acid (HCl, 37%), and hydrogen peroxide (H_2_O_2_, 30%), with known concentrations, were ordered from R&M, Malaysia, Selangor. Ammonia solution (NH₄OH, 25%) was obtained from Sigma Aldrich, Malaysia, Selangor. GO was synthesized via an improved Hummer′s method, as described in [58], where N-rGO was synthesized through a facile microwave-assisted hydrothermal method using ammonia solution as a nitrogen source.

### 3.2. Synthesis of GO

Briefly, 360 mL of H_2_SO_4_ and 10 mL of H_3_PO_4_ were mixed and stirred for 2.5 h in a 1 L round bottom flask. Then, 3 g of graphite powder and 18 g of potassium permanganate were added and mixed with prepared (H_2_SO_4_ + H_3_PO_4_) solution, which produced a dark green solution. The entire reaction was performed in an ice bath to avoid excessive heating and maintain the reaction temperature. The resulting solution was stirred for 2 h to exfoliate and oxidize the graphite flacks to few-layered graphene oxide. On completion of the oxidation reaction, hydrogen peroxide (H_2_O_2_) was added to avoid additional reactions. At this same point, the color of the resultant solution changed to dark yellow-brown. Afterwards, 1 L of deionized (DI) water was added to the solution to dilute it and adjust the pH level to ~1. The resultant mixture solution was then centrifuged at 7000 rpm to collect oxidized graphite flakes and to remove the acidic contents. The collected GO flake was further treated with 1 M HCl solution to eliminate impurities such as manganese and potassium ions, usually found in solids. Afterwards, the residual mixture was washed several times using DI water until the pH level exceeded ~1. Powder-like GO flakes were then extracted by centrifugation at 10,000 rpm. The GO particles were dry-frozen and finely ground into powder, referred to as GO flakes.

### 3.3. Synthesis of N-rGO

Initially, GO dispersion solution (75 mg/150 mL) was prepared in deionized water accompanied by 2 h constant stirring, followed by sonication of ~30 min. Subsequently, 5 mL ammonia solution was added to the resultant solution and continuously stirred for 12 h at 60 °C. The resulted solution was then poured into a 200 mL Teflon tube and heat-treated at 200 °C for 10 h in an autoclave machine using the oven. The collected N-rGO flakes were then exposed to microwave radiation using a microwave oven (Sharp, R735mt, 1 kW). The microwave intensity was adjusted to an optimum level to improve the reduction of oxygen-related functional groups and absorption of unreacted nitrogen contents. The N-rGO material samples were obtained at optimized parameters (time = 40 s and power = 800 W).

The resulting N-rGO sheets were washed several times with DI to extract the untreated nitrogen source. The dry N-rGO powder was eventually obtained through the dry freeze process. Subsequently, three samples of N-rGO, namely, N-rGO1, N-rGO2, and N-rGO3, were synthesized with three different concentrations of ammonia solution, 2, 5, and 10 mL, respectively.

### 3.4. Material Characterizations

GO and N-rGO sample material morphology was determined using FESEM (model 55 VP, Zeiss Supra, UK, Cambridge). Functional group analyses of GO and N-rGO powder samples were executed using KBr-FTIR (Fourier transform infrared spectrometer, Aquinox, 55-Bruker Instruments, Germany, Ettlingen). Functional groups in GO and N-rGO samples were also conformed through XPS spectral analysis using Origin software tools (Origin, Version 9, Waltham, MA, USA), Gaussian deconvolution and curve fitting of XPS multiple peaks were performed, wherein the baseline line estimations were also performed with the help of the adjacent averaging technique. The XPS spectra of GO and N-rGO samples were recorded using the XPS spectrometer (K-alpha, Thermo Scientific, Waltham, MA, USA), where the XPS spectrum for each sample was recorded (under pressure conditions 10^−8^ mbar) using a 1486.6 eV Al K-alpha radiation source from the range of 0 to 1200 eV. Crystallinity and interlayer distance: the X-ray diffraction (XRD) patterns were recorded for the GO and N-rGO powder samples to conform to the material’s crystallinity using X’Pert3-Powder, U.S.A., Westborough, wherein XRD patterns were recorded from 5 to 80° with a step size of 0.01 °/s. Imperfections and defects: the Raman spectra of GO and N-rGO samples were also recorded using the Raman spectrometer (Horiba Jobin Yvon HR-800 of Bruker Instruments, Germany, Ettlingen) to examine the defects and imperfections. 

Electrical conductivity: Hall effect measurements were conducted to confirm the enhanced conductivity of GO and N-rGO samples (using HMS, 3000 series, Phoenix, AZ, USA. Optical bandgap: UV/vis absorption characteristics of GO and N-rGO materials samples were also recorded using the UV/vis spectrometer (Agilent Technologies, Cary-100, Santa Clara, CA, USA), at ambient conditions.

### 3.5. Optical Bandgap Studies

The optical bandgap of synthesized material samples was obtained through UV/vis spectroscopy. NMP (N-Methyl-2-pyrrolidone) was used as a solvent to make an N-rGO-based dispersion solution, where an N-rGO dispersion solution with a concentration of 0.20 mg/mL was used to deposit thin films. Before a thin film deposition, a homogenous dispersion solution was obtained via ultrasonication of each material sample for 40 min, followed by magnetic stirring (1 h). Before thin film deposition, N-rGO-based dispersion solutions were centrifuged at 7000 rpm to separate the unexfoliated graphite structures from the dispersion solution to deposit uniform thin film. The POLOSTM spin coater was utilized to deposit thin film, where a uniform thin film of GO- and N-rGO-based samples was deposited on the glass substrate at 4000 rpm.

From a theoretical point of view, the correlation between parametric values was attained via UV/vis spectra, i.e., transmission (T), absorption (A), and reflectance (R), which may be established through Equation (2) [59].

(2) $$R+A+T=1\left(A=1-T-R\right)$$

The Beer–Lambert law, defined in Equation (3), defines a correlation between the light attenuation and certain material properties, at which the relationship between light absorbance and transmittance may be defined [32]. The absorption and transmittance coefficient can be calculated from Equations (4) and (5), respectively [60].

(3) $$I\left(d\right)=I_{o}e^{-ad}$$

(4) $$T={\left(1-R\right)}^{2}e^{-ad}$$

(5) $$a=\frac{2.303A}{d}$$

where *a* is the absorption coefficient, *I*(*d*) is the intensity at a depth of thickness *d*, and *I_o_* is the intensity at zero thickness. Moreover, the energy bandgap can also be deduced from absorption coefficients [61]. The optical bandgap (*E_g_*) can be calculated from Equation (6).

(6) $$E_{g}=hv-{\left(\frac{ahv}{\beta }\right)}^{\frac{1}{2}}$$

where *E_g_* is optical bandgap energy, *hv* is the photon energy, *a* is defined as the absorption coefficient, and β is the disorder parameter constant. Significantly, the nature of transitions is defined by j parametric values. Specifically, J parametric values, 1/2 describes the directly allowed transitions, 3/2 defines the directly forbidden transitions, 2 defines the indirectly allowed transitions, and 3 describes the indirect forbidden bandgap-related transitions [62]. Consequently, in this study, the optical bandgap for GO- and N-rGO-based samples was obtained with the help of Tauc plot calculation, i.e., *ahv*^2^ versus bandgap *hv*.

## 4. Conclusions

In this study, the facile synthesis of nitrogen-doped reduced graphene oxide was demonstrated using the microwave-assisted hydrothermal method. The successful synthesis of GO and N-rGO was confirmed by Raman, FTIR, XRD, XPS, FESEM, and EDX mapping and spectroscopic techniques. In XPS analysis of N-rGO, the maximum nitrogen doping concentration of ~5.51 at.% was conformed. In UV/vis spectroscopic analyses of GO and N-rGO, the tunable optical bandgaps were determined at about 3.4, 3.1, 2.5, and 2.2 eV, respectively. An enhanced electrical conductivity was observed in N-rGO samples, where an increment in electrical conductivity was observed with an increase in nitrogen doping concentration. More specifically, pyrrolic-N bonding configuration was found to be more dominant in N-rGO material samples, which enhance the π-bond in carbon atoms by reducing the Stone–Wales defect and forming percolation ways for electrons, which ultimately results in quitting a conduction gap and enhancing n-type electrical conductivity. The findings suggest that the synthesis approach adopted in the current study is suitable to produce N-rGO. The tunable optical bandgap in the visible region and enhanced n-type electrical conductivity (~0.781 S/cm) observed make it a promising material for developing future optical and electronic applications at the nanoscale.

## Figures and Tables

**Table 1:** The atomic percentage (at.%) of the elements found in the XPS analysis of GO and each N-rGO sample.

Material	C (at.%)	O (at.%)	N (at.%)
GO	65.78	34.23	-
N-rGO1	85.79	11.41	2.80
N-rGO2	83.97	11.15	4.53
N-rGO3	84.84	9.58	5.51

**Table 2:** The XPS analysis of N-rGO with each peak allocated to the specific functional group.

Peaks	Peak (eV)	Assignment
C 1s	284.2	C=C
	285.5	C–OH
	289.1	C=O
O 1s	532.2	C=O
	533.6	C–O
	535.8	C–OH
N 1s	398.1	Pyridinic-N
	400.3	Pyrrolic-N

**Table 3:** The interlayer distance and crystallite size obtained from the XRD analysis.

Sample	Interlayer Distance	Crystallite Size (L_c_)
GO	8.05 Å	29.75 nm
N-rGO1	4.26 Å	3.19 nm
N-rGO2	4.10 Å	3.55 nm
N-rGO3	4.05 Å	3.83 nm

**Table 4:** The Hall effect measurement of GO and N-rGO at 300 K.

S/No	Material	Conductivity S cm^−1^	Carrier Density/ cm^−3^	Hall Coefficient/ cm^3^ C^−1^	Charge Carrier Mobility cm^2^/(V.s)
1	GO	1.567 × 10^−6^ ± 2%	8.26 × 10^−9^	1.21 × 10^10^	1.960 × 10^−2^
2	N-rGO1	0.625 ± 2%	−0.118	8.421	0.214
3	N-rGO2	0.757 ± 2%	−0.093	10.676	0.252
4	N-rGO3	0781 ± 2%	−0.083	11.932	0.368

## Data Availability

Not applicable.

## Supplementary Materials

The following are available online. Figure S1: Raman deconvolution of N-rGO1 and N-rGO2 samples. Table S1: Percentage value of I peak and D″ peak.

## Sample Availability

Samples of the compounds are not available from the authors.
