# Supplementary material for: Effect of Nitrogen Doping on the Optical Bandgap and Electrical Conductivity of Nitrogen-Doped Reduced Graphene Oxide
Source: Molecules. 2021 Oct 25;26(21):6424. doi: 10.3390/molecules26216424 (PMC8588234; doi:10.3390/molecules26216424)
Supplement: Supplementary file 1 [file molecules-26-06424-s001.zip › molecules-1329615-supplementary.pdf]

Effect of Nitrogen Doping on the Optical Bandgap and Electrical Conductivity of Nitrogen-Doped Reduced Graphene Oxide

SUPPLEMENTARY NOTES

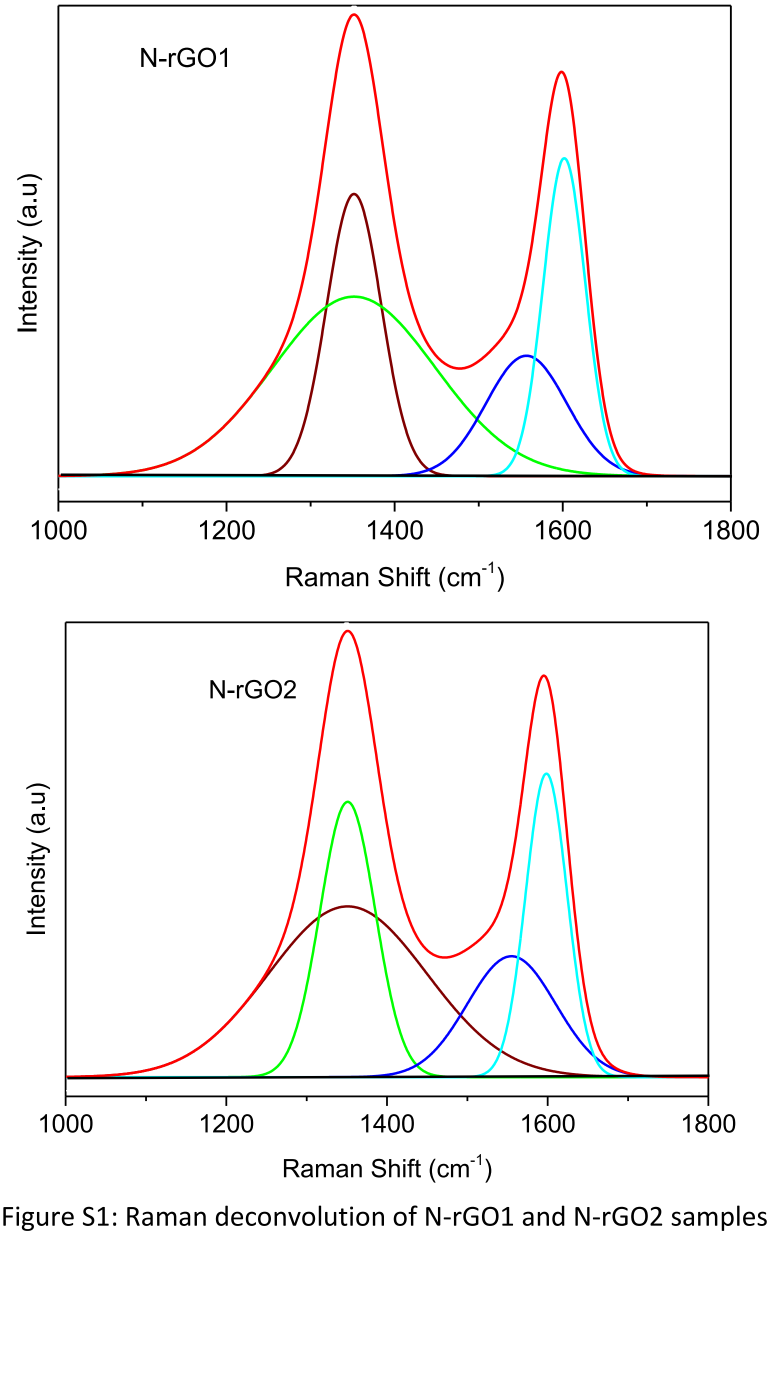

Figure S1: Raman deconvolution of N-rGO1 and N-rGO2 samples.

Table S1

**Sample**	**I peak (%)**	**D” peak (%)**
N-rGO1	52.5	12.25
N-rGO2	46.2	11.51
N-rGO3	43.7	10.35
